# Conceptualizing and measuring external context in implementation science: Studying the impacts of regulatory, fiscal, technological and social change

**DOI:** 10.1186/1748-5908-10-S1-A72

**Published:** 2015-08-20

**Authors:** Alison B Hamilton, Brian S Mittman, Alicia M Eccles, Craig S Hutchinson, Gail E Wyatt

**Affiliations:** 1 Department of Psychiatry and Biobehavioral Sciences, David Geffen School of Medicine, University of California, Los Angeles, CA, 90095, USA; 2 Center for the Study of Healthcare Innovation, Implementation and Policy (CSHIIP), VA Greater Los Angeles Healthcare System, Los Angeles, CA, 91343, USA; 3 Center for Implementation Practice and Research Support, VA Greater Los Angeles Healthcare System, Los Angeles, CA, 91343, USA; 4 Department of Research and Evaluation, Kaiser Permanente Southern California, Pasadena, CA, 91101, USA

## Introduction

Researchers in the fields of implementation and improvement science have increasingly recognized the importance of context in understanding implementation processes and outcomes (Damschroder et al, 2009; Taylor et al, 2011; Kaplan et al, 2012). To date much of the attention in conceptualizing and measuring contextual factors has targeted internal context, including features of the organizational settings in which improvement and implementation processes occur. The theoretical literature also highlights the importance of external context, however, comprising features of the external environment. We identified several features of external context influencing the progress and success of an ongoing NIMH-funded hybrid effectiveness-implementation study of an innovative HIV/AIDS prevention program (Hamilton et al, 2014). This abstract presents a preliminary taxonomy and definitions of key dimensions of external context and describes the role of this taxonomy in augmenting and elaborating existing frameworks for context in implementation and improvement science.

## Methods

This community-engaged partnership project is conducted by a multi-disciplinary team of university-based researchers and community agency staff. Weekly project calls are conducted to review and discuss progress in project recruitment, intervention delivery and evaluation. Specific barriers are identified and discussed during the weekly calls, and solutions proposed and implemented subsequently. We reviewed notes from these calls and notes from interviews with agency leaders to identify the key features of external context reported to be important influences on project progress.

## Results

Key dimensions of agency environments, or external context, identified in in this study include regulatory factors (e.g., changing regulations, expectations and programs offered by government agencies), fiscal factors (e.g., changes in healthcare and public health financing related to the Affordable Care Act and changes in insurance coverage and reimbursement policies and rates), technological change (including the introduction of Pre-Exposure Prophylaxis, PrEP, for HIV/AIDS), and social and economic change and pressures (e.g., the Great Recession and resulting pressures on low-SES clients and their motivation and ability to participate in prevention programs).

## Implications for D&I

Growth in the pace and magnitude of changes in the external environment and context of healthcare delivery and public health agencies highlight the need for richer, more complete conceptualization and measurement of these factors in implementation science. This study offers preliminary guidance in this development.

